# Formulation and Characterization of Epalrestat-Loaded Polysorbate 60 Cationic Niosomes for Ocular Delivery

**DOI:** 10.3390/pharmaceutics15041247

**Published:** 2023-04-14

**Authors:** Axel Kattar, Ana Quelle-Regaldie, Laura Sánchez, Angel Concheiro, Carmen Alvarez-Lorenzo

**Affiliations:** 1 Departamento de Farmacología, Farmacia y Tecnología Farmacéutica, I+D Farma Group (GI-1645), Facultad de Farmacia, Instituto de Materiales (iMATUS), and Health Research Institute of Santiago de Compostela (IDIS), Universidade de Santiago de Compostela, 15782 Santiago de Compostela, Spain; 2 Departamento de Zooloxía, Xenética y Antropoloxía Física, Facultade de Veterinaria, Universidade de Santiago de Compostela, 27002 Lugo, Spain; 3 Preclinical Animal Models Group, Health Research Institute of Santiago de Compostela (IDIS), 15706 Santiago de Compostela, Spain

**Keywords:** niosome, diabetic eye, HET-CAM, zebrafish, epalrestat, cationic lipid

## Abstract

The aim of this work was to develop niosomes for the ocular delivery of epalrestat, a drug that inhibits the polyol pathway and protects diabetic eyes from damage linked to sorbitol production and accumulation. Cationic niosomes were made using polysorbate 60, cholesterol, and 1,2-di-O-octadecenyl-3-trimethylammonium propane. The niosomes were characterized using dynamic light scattering, zeta-potential, and transmission electron microscopy to determine their size (80 nm; polydispersity index 0.3 to 0.5), charge (−23 to +40 mV), and shape (spherical). The encapsulation efficiency (99.76%) and the release (75% drug release over 20 days) were measured with dialysis. The ocular irritability potential (non-irritating) was measured using the Hen’s Egg Test on the Chorioallantoic Membrane model, and the blood glucose levels (on par with positive control) were measured using the gluc-HET model. The toxicity of the niosomes (non-toxic) was monitored using a zebrafish embryo model. Finally, corneal and scleral permeation was assessed with the help of Franz diffusion cells and confirmed with Raman spectroscopy. Niosomal permeation was higher than an unencapsulated drug in the sclera, and accumulation in tissues was confirmed with Raman. The prepared niosomes show promise to encapsulate and carry epalrestat through the eye to meet the need for controlled drug systems to treat the diabetic eye.

## 1. Introduction

In 2021 the prevalence of diabetes in adults ranging from 20 to 79 years worldwide was 9.8%, representing 536.6 million patients [1]. Diabetic patients have elevated levels of glucose in their blood, which leads to a range of conditions. A subcategory of complications derived from diabetes is diabetic ocular diseases. Examples include diabetic retinopathy, diabetic keratopathy, or cataracts, which have been linked to the polyol pathway [2]. These different illnesses affect the patient with loss of vision, culminating in blindness if left untreated. The current treatments for diabetic ocular diseases involve intravitreal injections [3], laser treatment [4], or even vitrectomy [5].

The polyol pathway transforms glucose into sorbitol when hexokinases active in the Embden-Meyerhof pathway are saturated, and subsequently, glucose is oxidated to sorbitol and further transformed into fructose. It is important to mention that this pathway is rarely used by the body in a healthy state as it is only activated at high intracellular glucose concentrations [6]. However, in the case of diabetes, the blood glucose levels can be high enough for the reaction balance to favor sorbitol production. Sorbitol is not permeable to cell membranes and accumulates inside the cells, and this excess creates osmotic stress [7]. Moreover, the conversion of glucose to sorbitol consumes NADPH which depletes NADPH stock [8] and competes with glutathione reduction, which in turn adds to oxidative damage [9]. This induces a lack of antioxidants and therefore increases the number of reactive oxygen species (ROS), increasing the oxidative stress in the affected tissue [10].

The polyol pathway can be blocked by inhibiting aldose reductase, an essential enzyme in the reduction of glucose to sorbitol [7,11]. In the case of diabetic retinopathy, the posterior segment of the eye is affected by sorbitol accumulation, and therefore, a carrier able to deliver the aldose reductase inhibitor to the inner eye tissues is needed. The aldose reductase inhibitor epalrestat is approved in Japan for oral administration thrice a day in 50 mg doses [12] to treat diabetic neuropathy. Its IC_90_ as an aldose reductase inhibitor was found to be 2.5 × 10^−2^ mg/mL [13] and an IC_50_ of 1.41 × 10^−6^ mg/mL in rat lens [14]. Epalrestat was shown to reduce aldose reductase expression and vascular endothelial growth factor (VEGF) secretion in retinal pigment epithelial cells [15]. Compared to methylcobalamine, epalrestat taken orally thrice daily for twelve weeks is better tolerated by patients and showed fewer adverse effects [16].

Although the information on ocular delivery systems of aldose reductase inhibitors is limited, topical instillation of tolrestat in rats was successful in preventing cataract development [17]. Biodegradable injectable implants for sustained delivery of N-4- (benzoylaminophenylsulfonyl glycine) also demonstrated inhibition of aldose reductase activity and VEGF expression in ARPE cells, as well as in galactose-fed rats [18]. Similarly, 2-methylsorbinil suspensions were shown to be successful in inhibiting cataracts in galactose-fed rats [19]. The only previous work attempting to deliver epalrestat to the posterior segment of the eye made use of contact lenses either through direct loading [20] or previous encapsulation in PEGylated solid lipid nanoparticles [21]. This work aims to show the viability of a nanoparticle system as a topical delivery system for epalrestat without the need for a contact lens.

In order to deliver epalrestat to the therapeutic site topically, it has to be encapsulated to cross different ocular barriers due to the high hydrophobicity of the molecule. Therefore, niosomes were prepared to encapsulate the drug and carry it to the posterior segment for it to reach the therapeutic site [22]. Niosomes are vesicles of small size with a bilayer made of surfactants and supplementary molecules that self-assemble through hydrophobic interactions as the involved molecules are amphiphilic. The shape, size, and surface charge can be modified by adjusting the concentration and molar ratios of different reagents [23,24]. They are being investigated for topical administration in ocular and skin tissue [25,26,27].

This work aims to formulate a niosomal carrier that fits the requirements to deliver epalrestat to the posterior segment of the eye through the scleral route. This was done by selecting polysorbate 60 (Tween 60), cholesterol, and 1,2-di-O-octadecenyl-3-trimethylammonium propane (DOTMA) and preparing niosomes with specific size, polydispersity, and surface charge. The obtained systems loaded with epalrestat were characterized in depth using various methods testing its physicochemical properties, encapsulation efficiency, release profile, permeation profile through the porcine cornea and sclera, ocular irritability potential, impact on blood glucose levels, and toxicity. Alternatives to animal testing were used according to the 3Rs principles: the HET-CAM [28] model for the ocular irritability potential, an extension of this model (Gluc-HET) to monitor the effect of the loaded niosome on the blood glucose levels [29], a zebrafish embryo model was used to assess the toxicity of the loaded niosomes [30,31,32], and porcine eye tissues from the slaughterhouse for ex vivo permeability tests.

## 2. Materials and Methods

*Materials.* Polysorbate 60 MW 1311.7 g/mol (Tween 60, HLB 14.9, Sigma Aldrich, Buchs, Switzerland), polysorbate 80 MW 1310 g/mol (Tween 80, HLB 15, Sigma Aldrich, Switzerland), 1,2-di-O-octadecenyl-3-trimethylammonium propane (chloride salt) (DOTMA, 670.58 g/mol) (Avanti, Alabaster, AL, USA), epalrestat (319.4 g/mol) (TCI, Tokyo, Japan), cholesterol (386.7 g/mol) (Chemtrec, Madrid, Spain), ethanol (VWR Chemicals, Briare, France), dichloromethane (Fischer Scientific, Waltham, MA USA), chloroform (Cienytech, Santiago de Compostela, Spain), phosphate-buffered saline (Life Tecnologies Co., Carlsbad, CA, USA), sodium chloride (Labkem, Barcelona, Spain), potassium chloride (Panreac, Castellar del Vallès, Spain), sodium bicarbonate (Merck, St Louis, MO, USA), calcium dihydrochloride (Merck, Darmstadt, Germany), potassium dihydrogen phosphate 1-basic (Panreac, Castellar del Vallès, Spain), disodium hydrogen phosphate dihydrate (VWR Chemicals, Briare, France), phosphate-buffered saline solution (Sigma-Aldrich, Lyon, France), Hanks’ Balanced Salt Solution (HBSS) (Paisley, Scotland, UK), glibenclamide (Roche, Basel, Switzerland). Ultrapure water (resistivity > 18.2 MΩ cm) was obtained by reverse osmosis (Milli-Q^®^, Millipore Ibérica, Madrid, Spain).

*Niosome formulation.* The protocol was adapted from previous reports [33,34,35]. Briefly, polysorbate 60 and cholesterol were dissolved in 2 mL ethanol in the presence or absence of DOTMA (at 1/0.42/0, 1/0.42/0.075 and 1/0.42/0.158 molar ratios of Tween 60/cholesterol/DOTMA as shown in Table 1). The combined total amount was kept at 76.38 µmol. Epalrestat (2 mg) was dissolved in 500 µL ethanol and added to the flask. The organic solvent was evaporated in a round bottom flask with a rotary evaporator at 70 °C under 50 mbar pressure to create a film. The film was subsequently desiccated for 30 min. 10 mL of ultrapure water was added to the flask. The film was removed from the walls of the flask by ultrasonication for 30 min. In order to form the niosomes, the solution was sonified for 90 s at 20% amplitude on a Branson Digital Sonifier 450 (Marshall Scientific, Hampton, NH, USA). This yielded epalrestat-loaded niosomes in water. In order to remove the unencapsulated drug, dialysis was performed for 30 min in 500 mL ultrapure water with 1 vol% Tween 80 with a 14,000 Da dialysis tubing (Sigma-Aldrich, Milwaukee, WI, USA).

The formulations were named with a code TCDX based on the included components: T for Tween, C for cholesterol, D for DOTMA, and x for the molar percentage of DOTMA present in the niosome. The code FD (free drug) was used to refer to a solution of 10% ethanol in water containing epalrestat at the same concentration as the epalrestat in the niosome formulation. For storage, the niosomes were kept in 15 mL Falcon tubes at room temperature in the absence of light.

*Niosome characterization.* The particle size and zeta potential of the niosomes were measured with DLS using a Zetasizer Nano (Malvern Instruments, Herrenberg, Germany) in ultrapure water at 20 °C with 10 s equilibration time using backscatter. The values taken were measured by intensity. The pH of the formulation solution was measured using a GL22 pH & ion meter (Crison, Barcelona, Spain). Viscosity at 35 °C was recorded between 0.05 and 200 rad/s in a Rheolyst AR-1000N rheometer (TA Instruments, Newcastle, UK) equipped with a Peltier plate and a cone geometry (40 mm diameter, 2°).

*Stability study.* The stability of epalrestat-loaded niosomes over 7 days was assessed at 4 °C (fridge) and 25 °C (oven) in the absence of light. The size, polydispersity index, zeta-potential, and content in epalrestat were measured at time points 0 and after 7 days. 

*TEM.* Drops of 5 µL of blank and epalrestat-loaded niosome dispersions were placed on carbon-coated grids, and the excess the solution was removed with filter paper. The samples were dyed with 1% phosphotungstic acid in water. The grid was allowed to dry and observed using a high-resolution JEM-1011 transmission electron microscope (JEOL USA Inc., Peabody, MA, USA).

*HPLC.* Quantitative analysis of epalrestat was performed on a Waters 717 plus Autosampler with a 4.6 × 250 mm C18 Symmetry column (Waters, Wexford, Ireland) with 5 µm pores. The mobile phase was acetonitrile:elution buffer 45:55 (*v*/*v*). The elution buffer was composed of 25 mM potassium dihydrogen phosphate and 25 mM disodium hydrogen phosphate dihydrate in ultrapure water adjusted to pH 6.5 with phosphoric acid. The flow rate was 0.85 mL/min, the detection wavelength was 295 nm, the temperature was maintained at 25 °C, and the injection volume was 40 µL. The calibration curve was prepared with concentrations ranging from 1 to 10 µg/mL with an increment step of 1 µg/mL. The retention time of epalrestat was 4.5 min. The HPLC quantification method was validated with regard to specificity, detection and quantitation limits, linearity, accuracy, precision, and range (Supplementary Material Figure S1).

*Encapsulation efficiency.* The encapsulation efficiency (*EE*%) was calculated by dialyzing the niosomes for 30 min in ultrapure water with 1 vol% Tween 80 [36] and analyzing the medium with HPLC. The dialysis membrane had a molecular weight cutoff of 12,000 Da and an effective dialysis area of 4.2 cm^2^. The concentration was confirmed by lysing the niosomes with methanol [34] and measuring the concentration of the drug encapsulated by HPLC. The efficiency was then calculated with Equation (1) [35]:

(1) $$EE\%=1-\frac{amount\;of\;drug\;out\;of\;the\;dyalisis\;membrane}{total\;amount\;of\;drug}*100$$

*Release study.* The release was tested by placing 5 mL of niosomes (0.20 ± 0.01 mg epalrestat/mL) in 14,000 MWCO dialysis tubing (Sigma-Aldrich, St Louis, MO, USA) and using 500 mL ultrapure water with 1% Tween 80 as receptor medium [36]. The medium was left at 20 °C for 20 days or 37 °C for 24 h stirring at 400 rpm with a magnetic stirrer. 1 mL of the medium was taken and replaced with 1 mL of fresh medium every day for 8 days and then every two days until day 21. The concentration of epalrestat in the release medium was quantified with HPLC. The niosomes left in the dialysis bag were lysed, and the remaining epalrestat was quantified with HPLC.

*HET-CAM.* For the Hen’s Egg Test on Chorioallantoic Membrane (HET-CAM) assay [28], fertilized eggs (15) were supplied by Coren (Ourense, Spain) and cleaned before incubation in a CCRS 0150 incubator (Ineltec, Tona, Spain) for 9 days at 37 °C and 60% relative humidity. On the day of the experiment, the shell of the eggs was pared off with a circular saw at the location of the air cell. The untouched inner membrane was moistened with a 0.9% NaCl solution, and the eggs were placed back in the incubator for 30 min. The 0.9% NaCl solution was subsequently removed, as well as the inner membrane, while being careful not to damage the blood vessels of the CAM underneath. Any non-viable egg was discarded. The positive control was NaOH 0.1M, and the negative control was 0.9% NaCl. The solutions tested were formulation TCD0, TCD5, and TCD10 in water loaded with 0.2 mg/mL epalrestat, unloaded niosomes of the same molar ratios, and epalrestat dissolved in ethanol: water 10/90 *v*/*v* mixture. The 300 µL of the testing solution was then added to the eggs, and the effect on the blood vessels regarding hemorrhage, lysis, and coagulation was recorded. The ocular irritability potential score was calculated with Equation (2) [28]:

(2) $$score=\frac{301-H}{300}*5+\frac{301-L}{300}*7+\frac{301-C}{300}*9,$$

with *H* = hemorrhage time (s), *L* = lysis time (s), *C* = coagulation time (s).

*Gluc-HET.* For the Gluc-HET assay [29], fertilized eggs (15) were supplied by Coren (Ourense, Spain) and cleaned before incubation in a CCRS 0150 incubator (Ineltec, Tona, Spain) for 11 days at 37 °C and 60% relative humidity. On the day of the experiment, the shell of the egg above the air pocket was pierced with a needle, and 300 µL of the testing solution was deposited inside the air compartment. The air compartment of the negative controls was pieced, but no solution was added [29]. The positive control was a solution of 0.002 mg/mL glibenclamide solution in HBSS. The eggshells are then closed off with parafilm. After 2 h incubation, the eggshell above the air compartment was removed, and the chorioallantoic membrane was cut next to a blood vessel with a scalpel. The blood vessel was placed on a flat metal tong and dried with paper. Once no moisture was absorbed anymore by the paper, the vessel was cut, and the blood glucose level was measured with a glucose meter (Contour next, Ascensia Diabetes Care, Basel, Switzerland).

*Zebrafish embryotoxicity test.* Epalrestat-loaded niosome toxicity was assessed using zebrafish embryos (*Danio rerio*) and the Fish Embryo Acute Aquatic Toxicity (FET) Test. Zebrafish embryos were selected around 3 h post-fertilization (hpf). The test was considered valid if the mortality of fish embryos was at least 30% in the positive control (3,4-dichloroaniline) and lower than 10% in the negative control. In our experiment, the mortality of the negative control was 3.3%, and the mortality of the positive control was 100%. The larvae were grown in autoclaved osmosis water. The experiment was carried out by including 5 or 10 µL of formulation in 200 µL of medium (4.9 µg/mL and 9.5 µg/mL, respectively) and quantifying the mortality at 24, 48, 72, and 96 h. The formulations tested were TCD0, TCD5, and TCD10. The experiments were conducted in triplicate.

*Corneal permeation.* Porcine eyes were supplied by a slaughterhouse and transported to the laboratory in diluted PBS solution at 4 °C in an ice bath. The corneas were dissected with 2–3 mm of surrounding tissue and washed with 0.9% NaCl to remove any attached tissue. The corneas were mounted in Franz diffusion cells with the outer part of the cornea facing up. The area available for permeation was 0.785 cm^2^. The receiving chamber was filled with 6 mL of Tween 80:water 10:90 *v*/*v* solution while making sure no bubbles formed and then agitated with a magnetic stirring rod at 400 rpm. The donor chamber was filled with 2 mL of carbonated buffer (pH 7.2) and closed off with parafilm to prevent evaporation. The system was then left to equilibrate for 1 h at 37 °C. Once the system was balanced, the carbonated buffer in the donor chamber was replaced by 2 mL of either 0.2 mg/mL epalrestat solution in 10 mL of ethanol:water 10:90 *v*/*v* or 0.2 mg/mL epalrestat encapsulated in the TCD0, TCD5, and TCD10 formulations. After 30 min, at 1 h and then every hour, 1 mL of the solution in the receiving chamber was removed and replaced with 1 mL of fresh Tween 80:water 10:90 *v*/*v* solution. After 6 h, the last sample was taken, and the corneas were incubated in ethanol at 37 °C for 24 h. They were then sonicated at 37 °C in an ultrasonic bath for 90 min. The resulting mixture was centrifuged at 1000 rpm at 25 °C for 5 min, and the supernatant was centrifuged at 14,000 rpm at 25 °C for 20 min. After filtration through 0.22 µm pore syringe filters (Scharlab, Barcelona, Spain), all the samples from the receptor chamber as well as the supernatant from the tissue incubation, were analyzed with HPLC according to the protocol described above. All experiments were carried out in triplicate (Supplementary Material Figure S2).

*Scleral permeation.* Scleral permeation was performed the same way as corneal permeation, except the tissue used to permeate through was the porcine sclera instead of the porcine cornea. 

*IR-Raman.* Porcine cornea and sclera were permeated with TCD0, TCD5, and TCD10 niosomes for 6 h under the same conditions as the corneal and scleral permeation experiment described above. The IR-Raman study was performed by taking a minimum of 3 points and a maximum of 6 points per cornea and per sclera (Supplementary Material Figures S3 and S4) and measuring the Raman scattering of the surface. Furthermore, a line scan was performed in the x-z plane (Supplementary Material Figures S5–S7). The excitation wavelength was 532.188 nm, the sample was kept at a temperature of 8 °C for the duration of the experiment, the laser power was 3 mW, and each point was measured with 60 accumulations, with an integration time of 0.3 s and an objective of ×50 (Zeiss LD EC Epiplan-Neofluar Dic 50×/0.55). The measurement was done on the top and bottom part of the tissue, and the absolute height of the peak (Supplementary Material Figure S8) (CCD cts) was compared between the top and bottom of each tissue. For the line scan, spectra were accumulated by taking 30 spectra per line and 15 lines per image, each spectrum at a distance of 1 µm from the previous point, both in the x and the z direction.

*Statistical analysis.* All conditions in the experiments were carried out in triplicate, and the data were shown as an average with a standard deviation when possible. Statistical analysis was carried out in Origin, making use of a one-way analysis of variance (one-way ANOVA) with Tukey’s comparison for evaluation. The difference between groups was statistically significant when the *p*-value was lower than 0.05.

## 3. Results

### 3.1. Niosome Characterization

Cationic niosomes were chosen to allow for efficient encapsulation [37], enhanced stability of the nanoparticles in suspension [38], increased ocular retention time [39], and increased bioavailability [40]. To prepare niosomes, the ratio of surfactant to helper lipids determined the stiffness and curvature of the bilayer. The incorporation of cholesterol changes the assembly of the bilayer as it lodges itself between the hydrophobic tails with the exception of its hydroxyl group [41,42]. In cell membranes, this translates into lowering the membrane permeability to water-soluble molecules, increasing the packing order of the lipids, reducing the bilayer fluidity, and separating the lipid tails to prevent crystallization [43,44,45].

The ratio of cholesterol incorporated in the niosomes affected the physicochemical properties as it intercalated itself within the organic chains of the surfactant in the niosome. The final mol fraction of cholesterol was determined by preparing and characterizing six formulations with different ratios of Tween 60 to cholesterol in terms of size, zeta- potential, and polydispersity index (Table 2). The short-term stability of the niosomes was assessed by running the same characterization experiments after 24 h. The size converged towards a similar size between 120 and 170 nm (Table 2). Formulations 5 and 6, which were outliers and had a hydrodynamic radius of 273.5 and 340.3 nm at t0, showed a decrease in size to 129.3 and 148.5 nm, respectively, after 24 h equilibration. The size of formulations 1 to 3 increased between 10 and 20 nm. Only formulation 4, containing a 0.5 mol fraction of cholesterol, stayed stable at the same size. The PDI remained stable while the zeta-potential rose across all samples. As the PDI of all formulations was similar, the initial greater size of formulations 5 and 6 could be due to the creation of multilamellar vesicles, which then evolved into smaller vesicles [46,47,48]. Due to the acceptable stability of formulation 3 with regards to size (+12 nm) and PDI (−0.03) while having similar zeta-potential stability (+2.84 mV) as all formulations, formulation 3 was chosen. It fitted our desired characteristics the most for cationic niosomes, having a size closest to 100 nm, a zeta-potential high enough to compensate with DOTMA incorporation, and a small size dispersion. The molar fraction of cholesterol was kept at 0.4 for all subsequent niosomal formulations.

**Figure 1:**
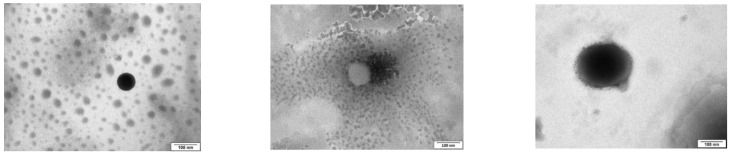
TEM images of epalrestat-loaded niosomes were used to assess the morphology of the niosomes (spherical) (from top to bottom: TCD0, TCD5, and TCD10). Scale bar 100 nm.

Niosomes with three different molar ratios of DOTMA were characterized in terms of size, zeta-potential, and PDI (Table 3, Figure 1). Increasing the DOTMA concentration decreased the size and polydispersity index while increasing the zeta potential. The pH of formulations was between 4.26 and 4.35. The encapsulation efficiency was between 99.22% ± 0.24 and 99.76% ± 0.35, and the drug loading was in the range of 25 to 26 mg epalrestat/g niosome for the three formulations.

Niosome dispersions behave as pseudoplastic (Supplementary Material Table S1) with a similar dependence on shear conditions. For a shear rate of 20 s^−1^, viscosity values of TCD0, TCD5, and TCD10 were 3.62, 4.23, and 3.32 mPa·s. Slightly high viscosity was recorded at very low shear rates, which can be related to the fact that relatively large niosomes offer resistance (although low) to start flowing. The obtained viscosity values were in good agreement with those recorded in previous reports [49,50].

The loaded niosomes were kept at 4 °C and 25 °C for 7 days and evidenced a slight increase in size and zeta-potential, while no significant changes were recorded in polydispersity index and encapsulation efficiency (Table 4). The niosomes were characterized again after two months at room temperature (Table 3). The size of the niosomes increased slightly while the PDI decreased slightly. The small decrease in the size of the niosomes when loaded with epalrestat may be due to the ability of the drug to disrupt the niosomal bilayer structure. Epalrestat is a hydrophobic drug that can partition into the hydrophobic region of the bilayer, causing the surfactants to rearrange themselves around the drug molecules. The rearrangement of the surfactants would lead to a slight reduction in the size of the niosomes [51,52]. The zeta-potential, however, increased except for the 10% DOTMA sample. The pH remained constant.

### 3.2. Epalrestat Release

**Figure 2:**
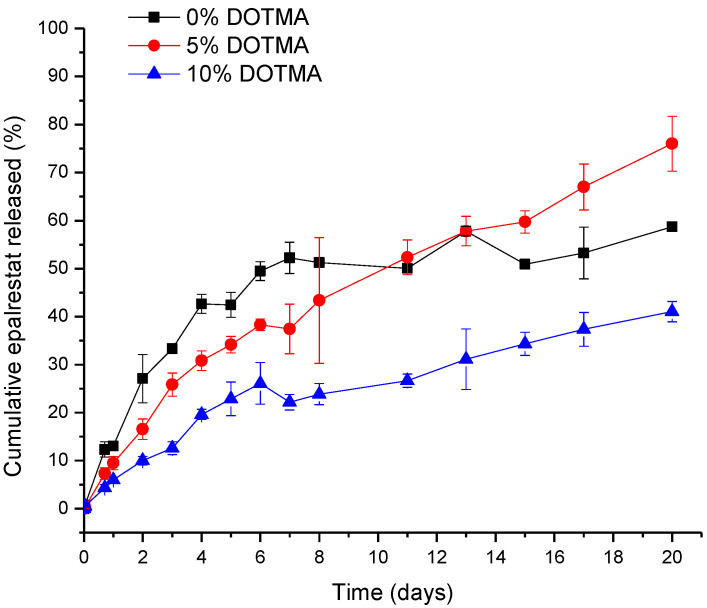
The release profile of epalrestat encapsulated in niosomes in Tween 80 1% aqueous medium at 20 °C over 20 days.

The release study was carried out in water containing 1% Tween 80 to ensure sink conditions, as epalrestat has a solubility in water of 0.047 mg/mL. With the results from the encapsulation efficiency, the final concentration of epalrestat was between 0.199 and 0.200 mg/mL in the dialysis bag, meaning that 100% epalrestat release corresponds to 1 mg released. The release profile of the niosomes over time is shown in Figure 2. In the first 8 days, epalrestat released from the niosomes negatively correlated with the DOTMA percentage of the niosome. However, after 10 days, 0% DOTMA niosomes showed a lower release rate, which may be related to its greater stability. These niosomes were the ones that changed their size during storage (Table 3), which indicates that they are less prone to destabilize. 

Another release experiment was carried out at 37 °C to mimic inside the back of the human eye. The amounts of released epalrestat over 24 h and non-released epalrestat are summarized in Table 5. The mass balance turned out to be about 96% for the niosomes, including DOTMA in their formulation, and 98.6% for niosomes without DOTMA. This finding indicated that niosomes protect epalrestat from degradation, which contrasts with the degradation of unencapsulated epalrestat in an aqueous medium [48]. This finding also confirms that niosomes are stable in the release medium.

### 3.3. HET-CAM Assay

**Figure 3:**
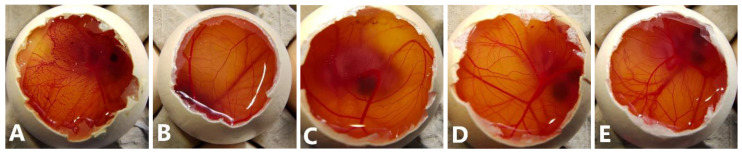
Pictures of the chorioallantoic membrane after 300 s (**A**): epalrestat dissolved in 10/90 ethanol/water (0.2 mg/mL), (**B**): unloaded TCD0 niosomes, (**C**–**E**): loaded niosomes (0.2 mg/mL) (**C**): TCD0, (**D**): TCD5, (**E**): TCD10.

The HET-CAM assay was performed using the three epalrestat-loaded niosomal formulations, one unloaded niosome formulation, and epalrestat dissolved in 10/90 *v*/*v* ethanol-water solution. The HET-CAM assay is not considered an animal experiment under Directive 2010/63/EU [53] as no nervous system is developed before day 11 of the embryo development. The positive (0.1 M NaOH) and negative controls (0.9% NaCl) had ocular irritability potential scores of 19.04 and 0, respectively. Epalrestat solution (0.2 mg/mL) (Figure 3A) triggered blood coagulation and had a score of 18.58. Differently, the loaded and unloaded niosomes did not show any noticeable hemorrhage of the blood vessels (Figure 3B–E) and obtained a score of 0. This indicated that the encapsulation of epalrestat decreases the ocular irritability potential and, therefore, allows for topical administration.

### 3.4. Gluc-HET Assay

**Figure 4:**
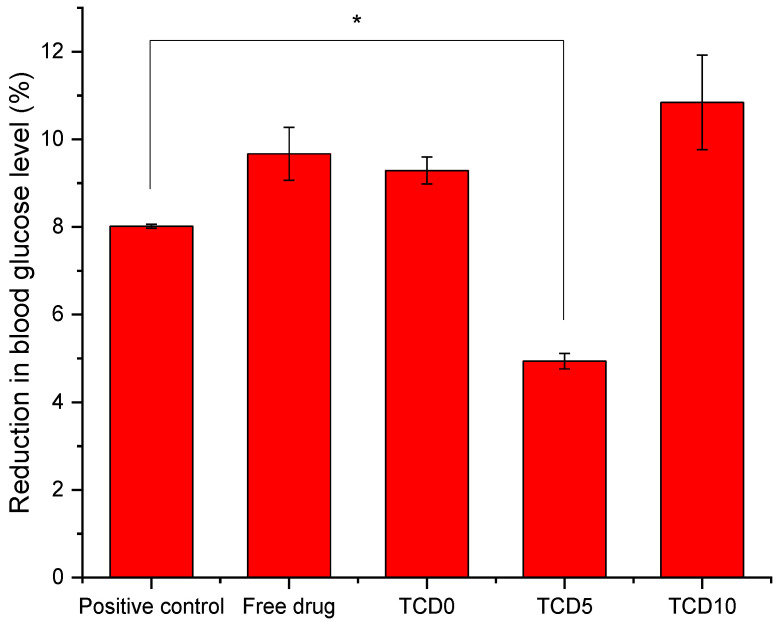
Blood glucose level after exposure to glibenclamide, epalrestat in 10/90 ethanol/water solution, TCD0, TCD5, and TCD10 niosomes loaded with epalrestat.* Statistically significant differences (*p* < 0.05).

To measure the effect on the blood glucose level, the gluc-HET [29] test was chosen as it presents a few advantages. It is not considered an animal experiment under Directive 2010/63/EU [53] as no nervous system is developed before day 11 of the embryo development. Furthermore, the embryos exhibit high glucose levels that are susceptible to insulin without interference from naturally produced insulin, which starts on day 12. Both the TCD0 and TCD10 loaded niosomal formulations behaved the same way as the epalrestat in solution (Figure 4) in that they reduced the blood glucose levels in a similar fashion to the positive control (glibenclamide). Tests carried out on the effect of the developmental stage on assay performance revealed a significant increase in the sensitivity of the embryos to the glucose-reducing compounds for day 10 and day 11 embryos [29]; therefore, day 11 was chosen to perform the experiment. Formulation TCD5 exhibited blood glucose level reduction but in a lower amount than the positive control.

### 3.5. Zebrafish Embryotoxicity Assay

Zebrafish embryotoxicity tests have been increasing in use for developmental toxicology and ToxCast high-throughput screening of chemicals and nanomaterials [54] due to the high concordance between zebrafish and mammalian studies demonstrating its potential to reduce and refine, if not replace animal studies [30,31,32]. The survival of *Danio rerio* (zebrafish) embryos was therefore measured after 96 h of exposure to loaded niosomes TCD0, TCD5, and TCD10 (Table 6).

With 5 µL solution, exposure formulation epalrestat-loaded niosomes were all highly compatible with the zebrafish embryos and demonstrated that the niosome encapsulation reduced the toxicity of epalrestat significantly.

### 3.6. Corneal and Scleral Permeation

**Figure 5:**
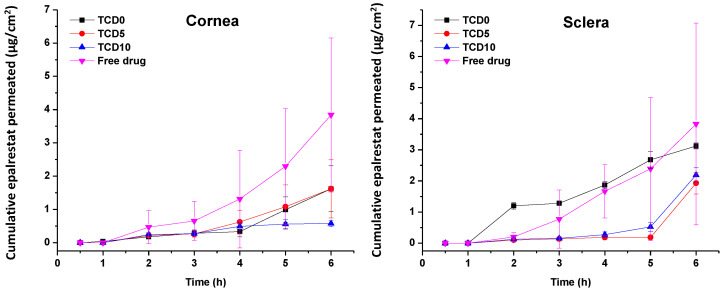
Amounts of encapsulated epalrestat permeated through the cornea (**left**) and sclera (**right**) after 6 h permeation experiment in Franz’s diffusion cells.

Epalrestat from formulations TCD0, TCD5, and TCD10 permeated at different rates through the porcine cornea and sclera (Figure 5). The steady-state flux and lag time were obtained from the slope and x-intercept of the linear regressions of the curves in Figure 5 (individual plots are shown in Figure S2, Supplementary Material) and used to calculate the permeability coefficient (Table 7) [55]. Encapsulated epalrestat permeation through the cornea was lower compared to epalrestat in solution (ANOVA; F3,12 = 9.32; *p* < 0.05). Differently, no statistically different results were recorded for permeability through the sclera for epalrestat in niosomes compared to free drug. Compared to the free drug solution, formulation in niosomes provided more reproducible data with less variability, and as expected, permeability coefficients through sclera were greater than through cornea, particularly in the case of the most cationic niosomes (TDC10) (Table 7).

**Figure 6:**
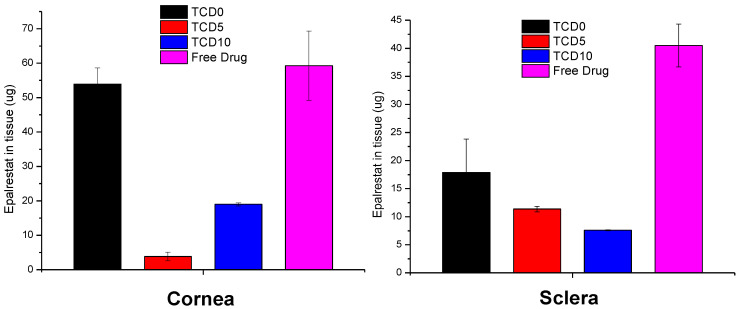
Epalrestat retained in corneal (**left**) and scleral (**right**) tissue after 6 h permeation experiment in Franz’s diffusion cells.

TCD5 and TCD10 niosomes displayed lower accumulation in corneal tissue than TCD0 niosomes and the epalrestat in solution. The niosomes showed lower drug accumulation than the epalrestat in solution in scleral tissue (Figure 6).

### 3.7. IR-Raman

**Figure 7:**
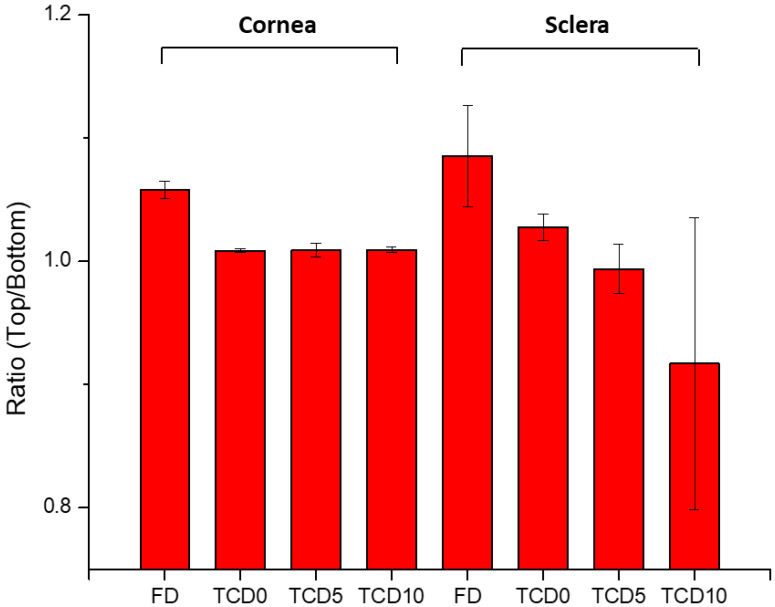
The ratio of the top-to-bottom intensity of Raman peaks indicates the presence of epalrestat in the tissue’s outside layer.

**Figure 8:**
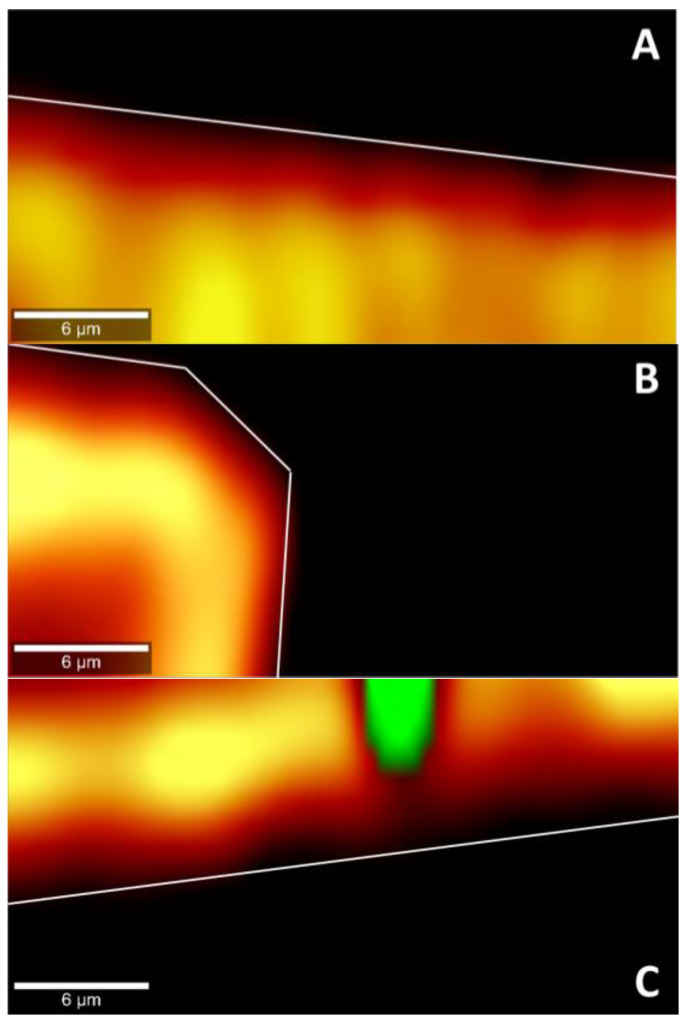
Cross-section heat map of epalrestat concentration in a 30 × 15 µm plane of the bottom of the cornea or the sclera after 6 h permeation of niosomes loaded with epalrestat (**A**): TCD0 cornea, (**B**): TCD10 cornea, (**C**): TCD10 sclera. Scale bar: 6 µm.

To confirm the permeation of epalrestat through the different tissues IR-Raman spectroscopy was performed on corneal and scleral porcine tissues after 6 h permeation experiments in Franz’s diffusion cells. The ratio of the Raman spectrum peak for epalrestat from the top part of the tissue (in contact with the donor chamber) to the bottom part of the tissue (in contact with the receiving chamber) was taken as an indication for confirmation of permeation of epalrestat through the tissue (Figure 7). Furthermore, pictures were assembled using accumulations of Raman spectra in a plane throughout the different tissues. This allowed for the production of heat maps showing the concentration of epalrestat at different levels of the tissue. Samples of TCD0 cornea, TCD10 cornea, and TCD10 sclera were able to produce readable pictures (Figure 8). This can be difficult due to the focus of the laser on the sample changing as the tissue moves with dehydration and burning. White lines were used to mark the tissue delimitation on the heat maps.

The ratio of top-to-bottom peak height was close to 1 for TCD0 cornea, TCD5 cornea, TCD10 cornea, and TCD5 sclera, indicating an equal presence of epalrestat on both sides of the tissue, while it is superior to 1 for FD cornea, FD sclera, and TCD0 sclera, indicating a higher presence of epalrestat on the side of the tissue in contact with the donor chamber than the side of the tissue in contact with the receiving chamber. TCD10 sclera is the outlier with a ratio lower than 1, which indicated a higher presence of epalrestat on the side of the tissue in contact with the receiver chamber. This indicates that permeation was confirmed in all tissues with various degrees.

In the pictures of cross sections of the tissue after permeation for 6 h with different epalrestat-loaded niosomal formulations, the heat map (the more yellow, the higher the concentration) represents the concentration of epalrestat in the tissue, while the green represents laser burning. Figure 8A,B showed the concentration of epalrestat from formulation TCD0 in the corneal tissue, with Figure 8A having channels of high concentration of epalrestat from formulation TCD10 in the z-orientation, and Figure 8B showing a bump in the tissue that got permeated both from the top and from the side. Finally, Figure 8C depicts the scleral tissue that suffered a burn due to laser exposure, represented in the green dot, which was explained by the higher susceptibility of the sclera to absorb light emitted by the laser and overheat. It, however, also showed less concentration gradient of epalrestat in the depth of the tissue. These findings go in the direction of the top-bottom ratio of samples TCD0 cornea, TCD10 cornea, and TCD10 sclera.

## 4. Discussion

To the best of our knowledge, this study is the first of its kind encapsulating epalrestat in niosomes and characterizing them in depth. Niosomes were designed by first choosing an adapted ratio of cholesterol to Tween 60, then testing the effect of the introduction of DOTMA to change the surface charge and render the niosomes cationic.

Three formulations were chosen, TCD0 as a control (−23 mV), TCD5 with low positive surface charge (+17 mV), and TCD10 as highly cationic (+40 mV). All three formulations exhibited sizes smaller than 100 nm, encapsulation efficiencies above 99%, spherical morphologies, and high stability over two months. When comparing the niosomes to other studies, the size of TCD0 niosomes was 20 nm bigger, and its polydispersity index was bigger than Tween 60/cholesterol prepared with the ethanol injection method [56]. The ethanol injection method is known to give more uniform niosomes [57] but is not appropriate for the encapsulation of all drugs. Two other studies made use of cationic niosomes and reported the size and zeta-potential, one substituting lycopene for cholesterol [33] and one substituting Tween 20 for Tween 60 [58]. In the first study, the size (101 nm) and the PDI (0.44) were bigger, and the zeta-potential (+33.8 mV) was lower than the TCD10 formulation. In the second study, the size (130 nm) was bigger, but the PDI (0.14) was smaller than the TCD10 formulation. Here the difference in non-ionic surfactant and helper lipid accounts for the difference in the physicochemical properties [41], as the preparation method was similar. The high encapsulation efficiency of epalrestat may be explained by the highly hydrophobic nature of epalrestat (water solubility of 0.0467 mg/mL [59]) that would tend to remain in the bilayer of the niosome, which is assembled through hydrophobic interactions. An encapsulating effect of hydrophobic molecules in the bilayer of the niosome was also seen in Span 80 niosomes with an added ionic surfactant [60], blends of Tween60/Span60 anionic niosomes, or Tween 80/Span80 PEGylated systems [61]. A complementary explanation is the length of the alkyl chains (stearyl C18), which usually leads to higher encapsulation efficiencies of hydrophobic cargo. Longer chains tend to display higher entrapment efficiency, with Tween 60 having a longer carbon tail than the lauryl chains (C12) of Tween 20 or Brij 35, for example [62,63]. With these encapsulation efficiencies, epalrestat concentration in the final niosome dispersion was close to 0.2 mg/mL. Niosome morphology (Figure 1) was spherical with low aggregation, and the size agreed with the DLS data. These images were in line with TEM images of reports describing niosomes made using the thin film hydration method [59].

The release of epalrestat from the niosomes reached 2.6%, 3.2%, and 4.0% after 12 h for the formulations TCD10, TCD5, and TCD0, respectively, which is around therapeutic levels for a tear film volume of 10 µL [13,14]. When releasing hydrophilic molecules, Tween 80/cholesterol niosomes displayed burst release behavior (90% in 1 h), as is the case with (−)-epigallocatechin gallate [52]. Release of lipophilic molecules also showed to be faster with mixed Span 60/Tween 60 cholesterol niosomes loaded with α-tocopherol (40% in 4 h) [64], as with Tween 60 niosomes containing meloxicam (90% in 7 h) [65]. However, it must be noted that these two examples are not exactly similar to our system as the α-tocopherol was released in a simulated gastric environment, and the niosomes containing meloxicam were micrometer size. Furthermore, the behavior of sustained release clashes with previous findings where epalrestat was incorporated into solid-lipid nanoparticles-laden lenses [21], and that showed a burst release. The epalrestat release looked similar to the sustained release obtained in lens delivery without nanoparticles after the initial release of adsorbed epalrestat [20].

To screen that the formulations are able to be used in eye drop format, alternatives to in vivo experimentation were used: ocular irritability potential assessment with the HET-CAM model, the effect on blood glucose levels using its variation of the gluc-HET assay, and the toxicity using a zebrafish embryotoxicity assay. In the HET-CAM model, the encapsulation of epalrestat decreased ocular irritability by lowering the irritability score of free epalrestat in solution from 18.6 to 0. This demonstrated the ability of niosomes to protect the eye from the irritability potential of epalrestat. In order to make sure that the introduction of this drug/carrier complex would not have a worsening effect on the blood glucose levels of a diabetic patient, it is important to make sure that the blood glucose levels remain the same when epalrestat-loaded niosomes are incorporated into the body. In our case, the niosomal formulation decreased the blood glucose levels at the same level as the positive control. The gluc-HET assay showed that all components lower the blood glucose levels in a similar regard as the positive control (−8 to −10%) with the exception of formulation TCD5. Formulation TCD5, however, still maintained blood glucose level reduction (−5%). These results point toward a possible increased efficacy in the context of diabetic treatment. However, as the mechanism in which these formulations lower blood glucose levels in the chorioallantoic membrane, further investigation is required before imagining epalrestat-loaded cationic niosomes as a glucose reduction agent. Finally, the encapsulation of epalrestat in niosomes increased the survivability of zebrafish embryos from 57.5% to 96% when exposed to 5 µL of the solution. Together these results support the potential use of formulations TCD0, TCD5, and TCD10 topically. When compared to other nanoparticles, such as zinc phthalocyanine and aluminum phthalocyanine liposomes, our niosomes performed similarly or better; the liposomes achieved between 100% and 65% or 85% survival rates, respectively [66]. Interestingly the results of zebrafish embryo survival with ionic-gradient liposomes go in the opposite direction, as the survival rate dropped when encapsulating bupivacaine in the liposomes [67]. This can be explained by the difference in the release rate of the drugs when encapsulated in niosomes or ionic-gradient liposomes, as the niosome would release epalrestat slower into the zebrafish larvae (2.5–4% for epalrestat against 65–100% for bupivacaine). Furthermore, a positive correlation between DOTMA percentage and mortality was observed (Table 6), which was to be expected as cationic nanoparticles are known to cause mortality in zebrafish embryos [68]. Therefore, it was expected that formulations TCD0 and TCD5 would be safer than TCD10.

The permeation of epalrestat from the niosomal formulations through the porcine sclera and the cornea was also monitored, reaching 2.5 µg/cm^2^ corneal permeation after 6 h in the case of TCD5. When compared to the study utilizing hydrogel lenses to deliver epalrestat [20], the amount of epalrestat accumulated in the tissues is higher using niosomes. However, it must be noted that our experiment took place on porcine cornea instead of bovine cornea, which is thinner. Also, in the previous study with epalrestat loaded in contact lenses, no drug was detected in the receptor after the 6 h test [20], which can be related to the lower drug concentration in the donor chamber that the contact lenses were able to supply. Indeed, in that previous study, the epalrestat control solution was 40 µg/mL, namely, five times lower than the control solution of free drug tested in our present study (200 µg/mL). That diluted free epalrestat solution facilitated the accumulation of only 10 µg drug in the bovine cornea [20], which is approximately 5 times less than the accumulation achieved in the present study. The results indicate that there is a therapeutic amount of epalrestat [14] that can be delivered to the cornea and the sclera with formulation TCD0. Further investigation needs to be done to confirm the accumulation of epalrestat in further tissues, but the results of our permeation experiments are positive as to the amounts of epalrestat able to traverse the cornea and sclera. The epalrestat steady-state flux obtained for the niosomal formulations is in the same order of magnitude as those recorded for skin formulations prepared with permeability enhancers [69]. The permeability coefficients recorded for epalrestat in niosomes were also in the range of that recorded for epalrestat solution in the mucosal-to-serosal transport through jejunum [70]. The lower permeability recorded for the most cationic niosomes through the cornea may be related to a higher binding to the cornea surface, which may hinder further diffusion of the niosomes and also of the drug encapsulated inside. Indeed, cationic liposomes have been demonstrated to prolong the retention time on the ocular surface [39]. Therefore, the charge density of niosomes should be the result of a compromise between the extended time of permanence on the ocular surface and efficient delivery of epalrestat to the inner eye tissues. It should also be noted that the niosomes have a certain thickening capability increasing 3 to 4 times the viscosity compared to the aqueous medium.

With the help of IR-Raman spectroscopy, drug permeation through the cornea and sclera was evidenced through the whole cross-sections of these tissues. A ratio of the Raman spectrum intensity taken from the top and bottom of the surface of the tissue equal 1, indicating that the concentration of epalrestat was similar on the tissue in contact with the donor as with the receiver chamber after 6 h of application of the formulation. The high values of the ratios of FD cornea (1.06) and FD sclera (1.09) can be explained by the faster access of unencapsulated epalrestat when it is free in solution. The staining of the tissue with the typical yellow color of epalrestat was also perceptible by the naked eye. The low value of the ratio for TCD10 sclera (0.92) is unusual but could be explained by interference from gasses liberated during the analysis process if the sample started to burn. Indeed, the peak of epalrestat situated at 1575 cm^−1^ is close to that of carbon (1581 cm^−1^) [71]. This also would explain the great variance in the results between the four measuring points. The heat maps of the cornea and sclera show the presence of epalrestat inside the tissue, permeating in different channels, adding weight to the results obtained from the Franz cell permeation experiments. While Raman spectroscopy has been used on ocular media for the detection of ganciclovir in ocular media in vivo [72] or for the detection of glutamate in tissues [73], this is, to our knowledge, the first time IR-Raman was used to confirm drug permeation through ocular tissues.

Epalrestat is a highly hydrophobic drug, and we found that it can be efficiently encapsulated in niosomes. The niosomes increase the drug’s apparent solubility and stability in water, which makes the preparation of eyedrops possible. Moreover, niosomes protect from the irritancy that a pure drug solution causes on the ocular surface. According to our results, the niosomes do not release epalrestat during storage but only when subjected to a strong dilution in the presence of other surfactant molecules, which may resemble the conditions on the ocular surface. The formulation TCD5 seemed to be the most balanced, as it was cationic, had sustained release of epalrestat over a long period, was safe for the ocular surface, and permeated both the cornea and the sclera. The capability to sustain drug release in vitro means that most part of the drug dose remains in niosomes when topically placed on the eye, and niosomes have been reported to enhance drug permeability in vivo [24]. Some previous reports on drug-loaded niosomes for ocular delivery have also shown in vitro sustained release in the frame of a few days for hydrophobic drugs such as fluconazole [74] and natamycin [75] or even mid-polarity drugs such as vancomycin [76], doxycycline [77] or naltrexone [78]. In vivo, studies evidenced the success of the niosome approach compared to the free drug solution, indeed probably due to the more sustained release. Niosomes have been shown to remain on the ocular surface longer than the drug solution. Thus, a sustained release may prevent a very rapid washout from the ocular surface while still creating a drug concentration gradient that facilitates the diffusion through eye tissues [24].

## 5. Conclusions

Our study demonstrated the ability of cationic niosomes to encapsulate epalrestat. Neutral to mild-cationic niosomal formulations showed suitable physicochemical characteristics for topical ocular drug delivery, supported by a low ocular irritability potential, high biocompatibility, sustained release, and permeability through the cornea and scleral tissue. Compared to epalrestat-loaded contact lenses or free drug solutions, niosomes can encapsulate more drugs, increasing apparent solubility, and protect better the drug from premature degradation while promoting the pass towards inner eye tissues. Moreover, as revealed in the HET-CAM and zebrafish embryotoxicity assays, drug encapsulation in niosomes makes the formulation safe. Similar concentrations of epalrestat in solution would be harmful to patients. These findings point out epalrestat-loaded niosomes as suitable for non-invasive drug delivery to inner eye structures.

## Figures and Tables

**Table 1:** Mol fractions of the surfactant, helper lipid, and cationic lipid are used to prepare the niosomes.

Formulation	Tween 60 (mol Fraction)	Cholesterol (mol Fraction)	DOTMA (mol%)
1	1	0.2	0
2	1	0.3	0
3	1	0.4	0
4	1	0.5	0
5	1	0.75	0
6	1	1	0
TCD0	1	0.4	0
TCD5	1	0.4	5
TCD10	1	0.4	10

**Table 2:** Size, PDI, and zeta-potential of Tween 60/cholesterol niosomes at t = 0 and t = 24 h were used to determine the molar fraction of cholesterol used in subsequent experiments.

Formulation	Time t = 0			Time t = 24 h		
	Size (nm)	PDI	Zeta-Potential (mV)	Size (nm)	PDI	Zeta-Potential (mV)
1	155.0 ± 53.3	0.51 ± 0.02	−14.53 ± 0.32	172.3 ± 52.9	0.55 ± 0.11	−12.11 ± 0.84
2	162.3 ± 63.2	0.54 ± 0.02	−19.83 ± 0.26	174.0 ± 34.2	0.47 ± 0.11	−15.16 ± 0.51
3	108.8 ± 13.4	0.58 ± 0.14	−17.82 ± 0.48	120.8 ± 12.7	0.55 ± 0.13	−14.98 ± 1.29
4	158.6 ± 62.6	0.60 ± 0.14	−24.43 ± 0.38	154.2 ± 30.6	0.54 ± 0.05	−21.79 ± 1.75
5	273.5 ± 59.7	0.57 ± 0.02	−24.04 ± 0.26	129.3 ± 13.8	0.47 ± 0.05	−20.21 ± 0.22
6	340.3 ± 71.8	0.54 ± 0.03	−24.64 ± 0.37	148.5 ± 25.0	0.55 ± 0.08	−23.88 ± 0.70

**Table 3:** Size, PDI, zeta-potential of epalrestat loaded Tween 60/cholesterol niosomes with 0, 5, and 10 mol% DOTMA at time 0 and after 2 months at room temperature.

Formulation	Time t = 0			Time t = 2 Months		
	Size (nm)	PDI	Zeta-Potential (mV)	Size (nm)	PDI	Zeta-Potential (mV)
TCD0	84	0.54	−23.34 ± 5.34	91	0.52	+1.75 ± 5.77
TCD5	68	0.46	+17.27 ± 10.29	93	0.36	+31.80 ± 7.58
TCD10	75	0.28	+40.39 ± 10.29	110	0.26	+42.07 ± 8.26

**Table 4:** The difference in size, PDI, zeta-potential, and drug content of epalrestat loaded Tween 60/cholesterol niosomes with 0, 5, and 10 mol% DOTMA after 7 days in storage at 4 °C and 25 °C.

Formulation	Δ Size (nm)		Δ Zeta-Potential (mV)		Δ Polydispersity Index		Δ Encapsulation Efficiency (%)	
	4 °C	25 °C	4 °C	25 °C	4 °C	25 °C	4 °C	25 °C
TCD0	22.1	26.3	−2.3	−3.6	−0.14	0.13	−1.2	−0.1
TCD5	33.0	59.1	11.1	7.9	−0.27	0.08	−1.1	−0.2
TCD10	17.9	14.6	21.4	26.3	−0.05	0.01	−0.7	−0.4

**Table 5:** Amounts of epalrestat released and remaining in the niosomes after 24 h in Tween 80 1% aqueous medium at 37 °C.

Formulation	Epalrestat Released after 24 h (%)	Epalrestat Remaining in the Niosomes (%)	Sum of Released and Non-Released Epalrestat (%)
TCD0	12.9 ± 0.50	85.7 ± 0.82	98.6 ± 0.66
TCD5	11.7 ± 0.33	84.3 ± 1.42	96.0 ± 0.87
TCD10	10.3 ± 0.53	86.2 ± 0.97	96.5 ± 0.75

**Table 6:** Survivability of zebrafish embryos after 96 h of exposure to different loaded niosome formulations.

Tested Formulation	Survival of *Danio rerio* (%)	
	5 µL Solution Exposure	10 µL Solution Exposure
Negative control	98.0	96.7
TCD0	98.0	56.7
TCD5	93.0	60.0
TDC10	96.6	43.3
Epalrestat in solution	57.5	40.0

**Table 7:** Steady-state flux, lag time, and permeability coefficient of different tissues and formulations.

Sample		Steady State Flux (µg/cm^2^ × h)	Lag Time (min)	Permeability Coefficient (×10^6^ cm/s)
Cornea	Free drug	1.029 (0.356)	152 (66)	1.43 (0.49)
	TCD0	0.469 (0.204)	163 (12)	0.65 (0.28)
	TDC5	0.453 (0.295)	138 (36)	0.63 (0.40)
	TDC10	0.095 (0.008)	33 (32)	0.13 (0.01)
Sclera	Free drug	0.887 (0.758)	118 (4)	1.23 (1.09)
	TCD0	0.634 (0.035)	57 (16)	0.88 (0.05)
	TDC5	0.538 (0.110)	201 (6)	0.75 (0.15)
	TDC10	0.636 (0.070)	196 (4)	0.88 (0.09)

## Data Availability

The data used to support the findings of this study are available in the Supplementary Material and from the corresponding author upon request.

## Supplementary Materials

The following supporting information can be downloaded at: https://www.mdpi.com/article/10.3390/pharmaceutics15041247/s1, Reference [79] are cited in the supplementary materials.
