# Supplementary material for: Formulation and Characterization of Epalrestat-Loaded Polysorbate 60 Cationic Niosomes for Ocular Delivery
Source: Pharmaceutics. 2023 Apr 14;15(4):1247. doi: 10.3390/pharmaceutics15041247 (PMC10142600; doi:10.3390/pharmaceutics15041247)
Supplement: Supplementary file 1 [file pharmaceutics-15-01247-s001.zip › pharmaceutics-2274664-supplementary.pdf]

Supplementary Material

HPLC method validation.

The niosomes were tested in duplicate and each release experiment data set was analyzed in separate. The validation of the HPLC method involved detection and quantitation limits, linearity, accuracy, and precision.

The limit of detection (LOD) and limit of quantitation (LOQ) are defined by[79]:

$$LOD = X_{b1} + 3S_{b1} \quad (3)$$

$$LOQ = X_{b1} + 10S_{b1} \quad (4)$$

The LOD and LOQ were found to be at 0.00813 µg/mL and 0.01896 µg/mL, respectively. **Error! Reference source not found.** shows the validation of the HPLC quantification. The validation method has been carried out at multiple points in time.

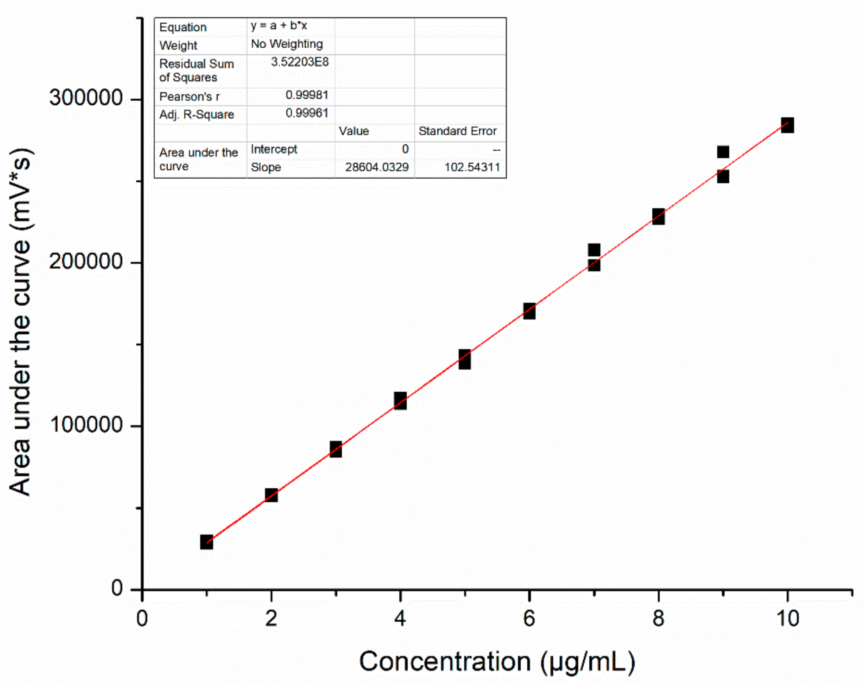

Figure S1. HPLC validation curve of epalrestat detection using the experimental conditions described in the HPLC section of the materials and methods.

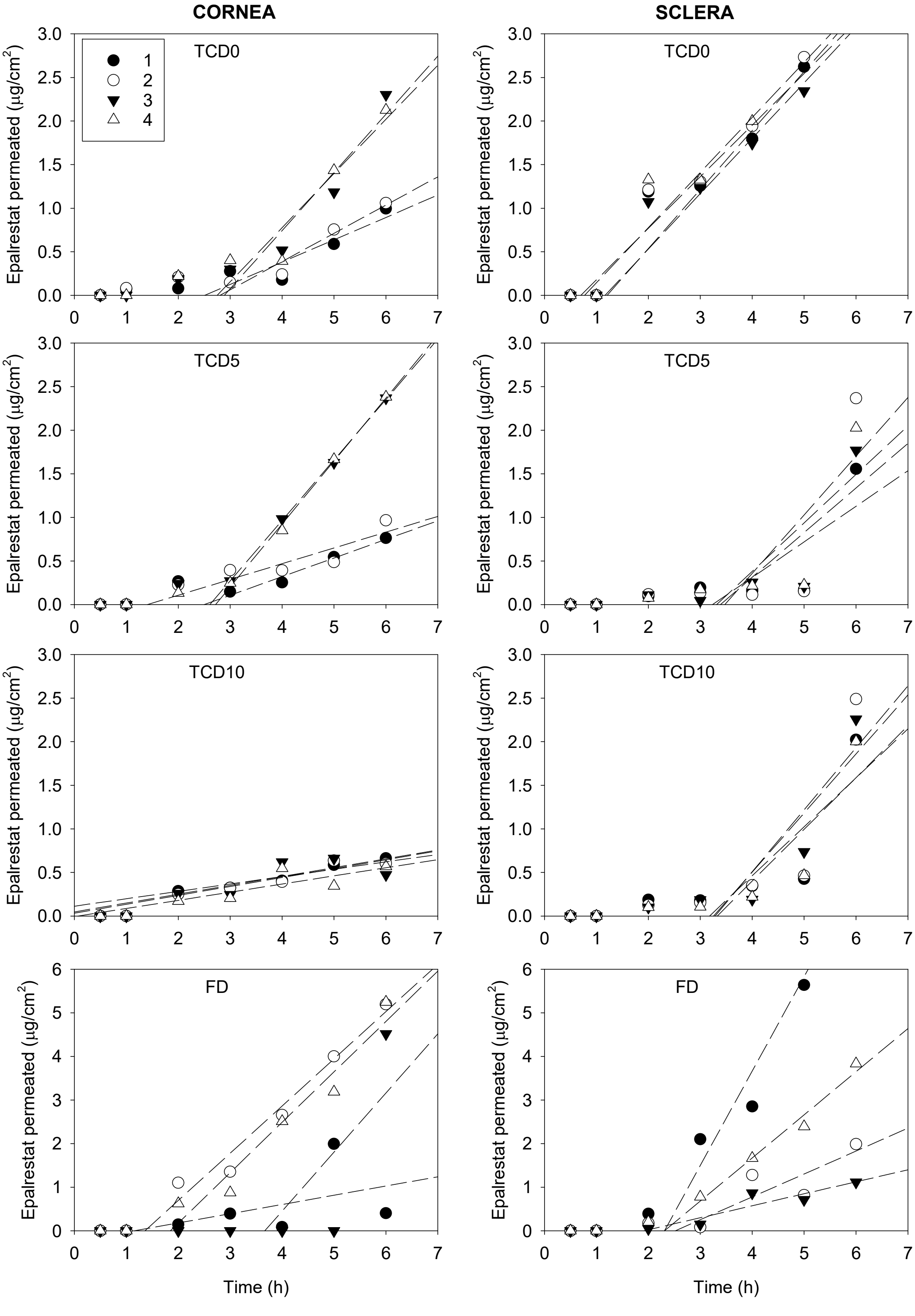

Figure S2. Epalrestat permeability data through cornea and sclera recorded for four independent replicates of each niosome formulation (TCD0, TCD5, TCD10) and control epalrestat solution (200 μg/mL).

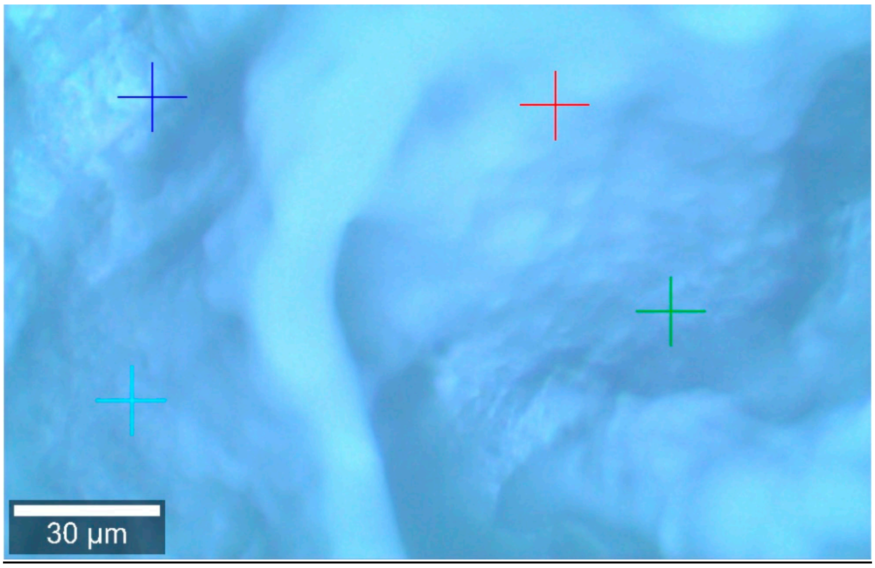

Figure S3. Image of the sample free drug cornea top under x50 magnification, with the crosses indicating the locations the Raman spectrum was taken.

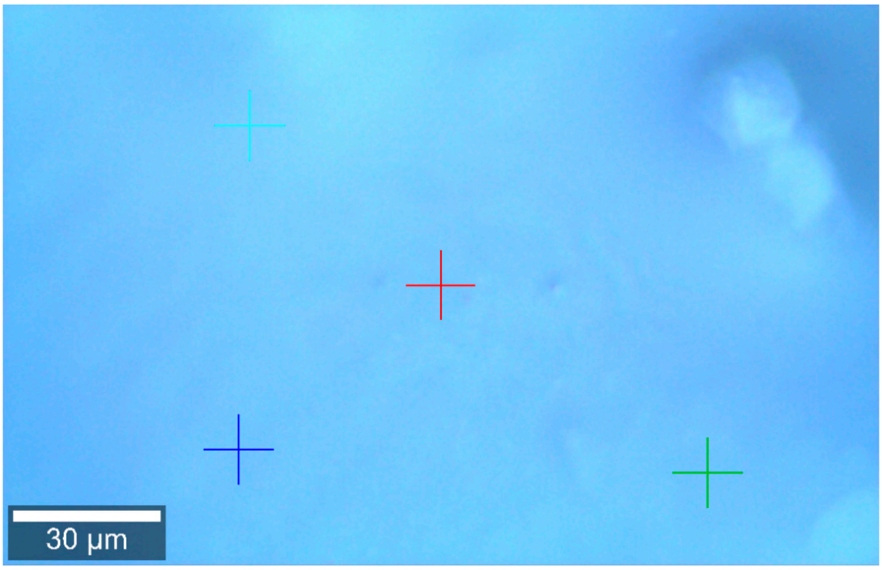

Figure S4. Image of the sample free drug cornea bottom under x50 magnification, with the crosses indicating the locations the Raman spectrum was taken.

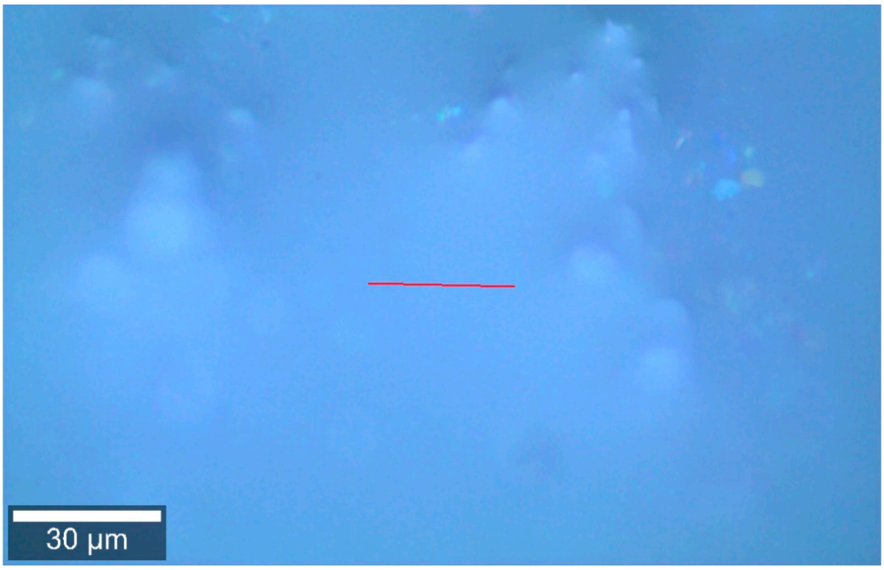

Figure S5. Image of the sample TCD0 cornea bottom under x50 magnification, with the line indicating the locations the Raman spectra were taken.

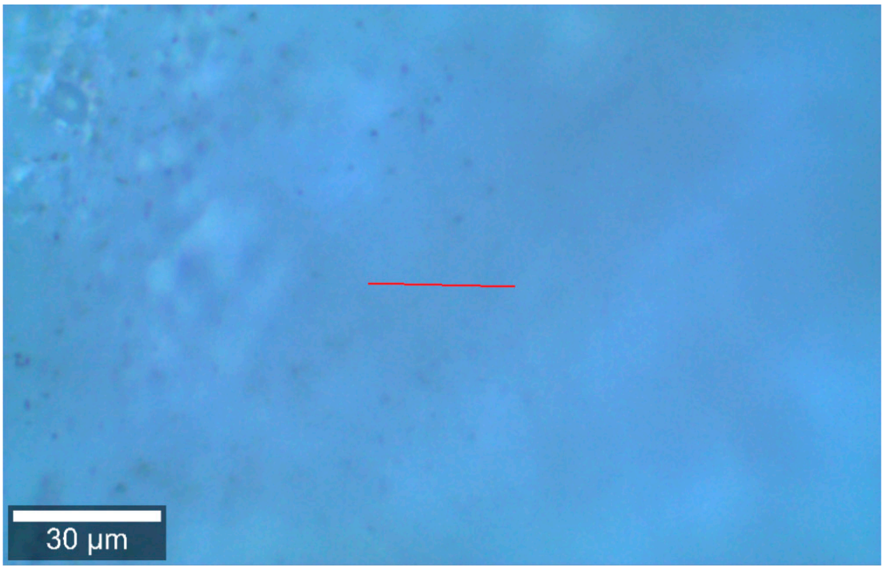

Figure S6. Image of the sample TCD5 cornea bottom under x50 magnification, with the line indicating the locations the Raman spectra were taken.

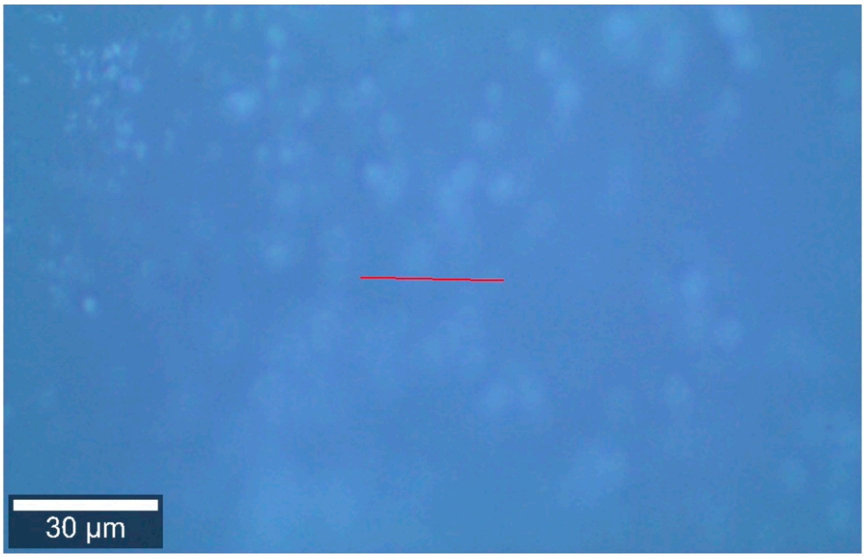

Figure S7. Image of the sample TCD10 sclera bottom under x50 magnification, with the line indicating the locations the Raman spectra were taken.

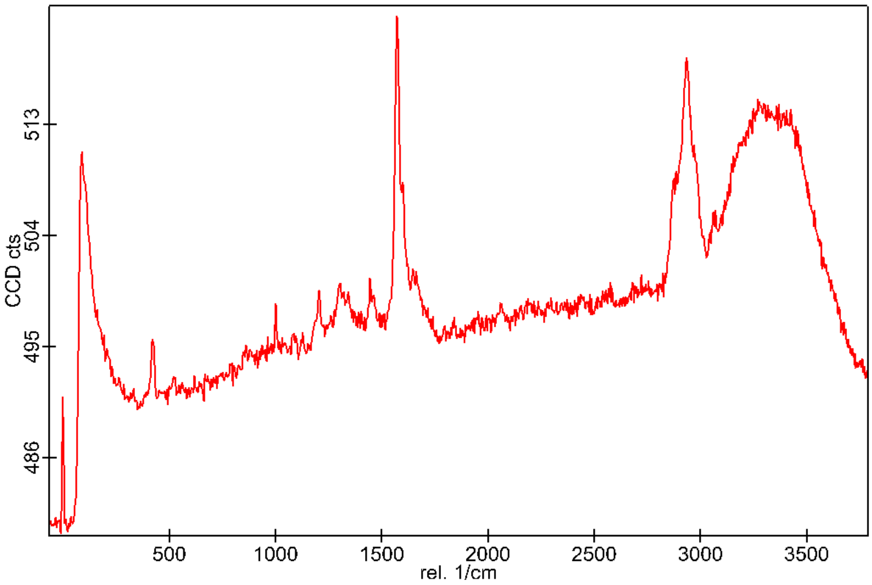

Figure S8. Raman spectrum of sample free drug cornea top at the blue cross in Figure A3.

Table S1. Viscosity values recorded at 35 ºC for the epalrestat-loaded niosomes under increasing shear stress conditions.

TCD0			TCD5			TCD10		
Shear stress (Pa)	Shear rate (s^{-1})	Viscosity (Pa·s)	Shear stress (Pa)	Shear rate (s^{-1})	Viscosity (Pa·s)	Shear stress (Pa)	Shear rate (s^{-1})	Viscosity (Pa·s)
0.05532	3.667	0.01508	0.06584	3.641	0.01808	0.04964	3.424	0.0145
0.05856	7.664	7.64E-03	0.08584	7.64	0.01124	0.05635	7.422	7.59E-03
0.06467	11.66	5.54E-03	0.05987	11.64	5.14E-03	0.0571	11.42	5.00E-03
0.04693	15.67	3.00E-03	0.04123	15.64	2.64E-03	0.05781	15.42	3.75E-03
0.07116	19.66	3.62E-03	0.08314	19.64	4.23E-03	0.06444	19.42	3.32E-03
0.05301	23.66	2.24E-03	0.05154	23.63	2.18E-03	0.06574	23.42	2.81E-03
0.07594	27.66	2.75E-03	0.0685	27.64	2.48E-03	0.07211	27.42	2.63E-03
0.08408	31.66	2.66E-03	0.09397	31.64	2.97E-03	0.07641	31.42	2.43E-03
0.08446	35.66	2.37E-03	0.09725	35.63	2.73E-03	0.07975	35.42	2.25E-03
0.08825	39.66	2.23E-03	0.1009	39.63	2.55E-03	0.0842	39.42	2.14E-03
0.0951	43.66	2.18E-03	0.1059	43.63	2.43E-03	0.0878	43.41	2.02E-03
0.09892	47.66	2.08E-03	0.09583	47.64	2.01E-03	0.09117	47.41	1.92E-03
0.08522	51.66	1.65E-03	0.07739	51.63	1.50E-03	0.0941	51.41	1.83E-03
0.1034	55.65	1.86E-03	0.1159	55.62	2.08E-03	0.1007	55.41	1.82E-03
0.09611	59.66	1.61E-03	0.08602	59.63	1.44E-03	0.1021	59.41	1.72E-03
0.1154	63.65	1.81E-03	0.1254	63.63	1.97E-03	0.1083	63.41	1.71E-03
0.1069	67.65	1.58E-03	0.1148	67.62	1.70E-03	0.111	67.41	1.65E-03
0.1015	71.65	1.42E-03	0.09972	71.62	1.39E-03	0.1137	71.41	1.59E-03
0.1076	75.65	1.42E-03	0.1007	75.62	1.33E-03	0.1173	75.4	1.56E-03
0.1092	79.65	1.37E-03	0.105	79.62	1.32E-03	0.1214	79.41	1.53E-03
0.1174	83.65	1.40E-03	0.1225	83.62	1.47E-03	0.1258	83.4	1.51E-03
0.1359	87.64	1.55E-03	0.149	87.62	1.70E-03	0.1342	87.4	1.54E-03
0.1323	91.65	1.44E-03	0.1274	91.63	1.39E-03	0.1348	91.4	1.48E-03
0.1328	95.64	1.39E-03	0.1418	95.61	1.48E-03	0.1387	95.4	1.45E-03
0.1377	99.65	1.38E-03	0.1318	99.63	1.32E-03	0.1423	99.41	1.43E-03
0.1522	103.6	1.47E-03	0.1651	103.6	1.59E-03	0.1513	103.4	1.46E-03
0.1454	107.6	1.35E-03	0.1533	107.6	1.43E-03	0.1505	107.4	1.40E-03
0.1419	111.6	1.27E-03	0.1424	111.6	1.28E-03	0.1523	111.4	1.37E-03
0.1448	115.6	1.25E-03	0.144	115.6	1.25E-03	0.1554	115.4	1.35E-03
0.1531	119.6	1.28E-03	0.1575	119.6	1.32E-03	0.1603	119.4	1.34E-03
0.1687	123.6	1.36E-03	0.1819	123.6	1.47E-03	0.1711	123.4	1.39E-03
0.1713	127.6	1.34E-03	0.1753	127.6	1.37E-03	0.1756	127.4	1.38E-03
0.1597	131.6	1.21E-03	0.1567	131.6	1.19E-03	0.1715	131.4	1.31E-03
0.1823	135.6	1.34E-03	0.1924	135.6	1.42E-03	0.1862	135.4	1.38E-03
0.1725	139.6	1.24E-03	0.1766	139.6	1.27E-03	0.1796	139.4	1.29E-03
0.1708	143.6	1.19E-03	0.1654	143.6	1.15E-03	0.1869	143.4	1.30E-03
0.1849	147.6	1.25E-03	0.1875	147.6	1.27E-03	0.1972	147.4	1.34E-03
0.1938	151.6	1.28E-03	0.2019	151.6	1.33E-03	0.2048	151.4	1.35E-03
0.1956	155.6	1.26E-03	0.2028	155.6	1.30E-03	0.2084	155.4	1.34E-03
0.1898	159.6	1.19E-03	0.1882	159.6	1.18E-03	0.2087	159.4	1.31E-03
0.1885	163.6	1.15E-03	0.1863	163.6	1.14E-03	0.2041	163.4	1.25E-03
0.213	167.6	1.27E-03	0.2286	167.6	1.36E-03	0.2169	167.4	1.30E-03
0.1975	171.6	1.15E-03	0.1967	171.6	1.15E-03	0.2212	171.4	1.29E-03
0.2211	175.6	1.26E-03	0.236	175.6	1.34E-03	0.2264	175.4	1.29E-03
0.2039	179.6	1.14E-03	0.2024	179.6	1.13E-03	0.2218	179.4	1.24E-03
0.2104	183.6	1.15E-03	0.2114	183.6	1.15E-03	0.2377	183.4	1.30E-03

0.2224	187.6	1.19E-03	0.2323	187.6	1.24E-03	0.2469	187.4	1.32E-03
0.2282	191.6	1.19E-03	0.2401	191.6	1.25E-03	0.2525	191.4	1.32E-03
0.2254	195.6	1.15E-03	0.2319	195.6	1.19E-03	0.2566	195.4	1.31E-03
0.2449	199.2	1.23E-03	0.2616	199.2	1.31E-03	0.25	199	1.26E-03
